# Boric Acid in Goiter

**Published:** 1902-03

**Authors:** 


### Boric Acid in Goiter.

---

In *American Medicine,* February 8th, D. Braden Kyle, M. D., gives a preliminary account of his experience with the internal administration of boric acid in eleven cases of goiter in which the enlargement of the thyroid was due to increase in its cellular elements and not to distended vessels, a septic condition, or new growth. In 1895 he observed that a goiterous patient who had been taking boric acid for some other condition improved rapidly in general health, and that the goiter became much smaller. He explains this on the theory that goiter is caused by the precipitation under certain conditions of a material within the body (due to perverted chemic reaction) which has a selective action upon the thyroid gland, stimulating its blood supply, etc. Boric acid he believes either corrects the faulty secretion or serves to neutralize the material precipitated or formed by the faulty chemistry of the secretion.

Dr. Kyle's treatment of the eleven cases was correction of any deficiency in the organs of elimination and the administration of ten to fifteen grains of boric acid in capsule form with a full glass of water every three hours. The reduction in the size of the gland occurred from simple atrophy. R.

